# cAMP/PKA Pathways and S56 Phosphorylation Are Involved in AA/PGE_2_-Induced Increases in rNa_V_1.4 Current

**DOI:** 10.1371/journal.pone.0140715

**Published:** 2015-10-20

**Authors:** Hua Gu, Yan-Jia Fang, Dong-Dong Liu, Ping Chen, Yan-Ai Mei

**Affiliations:** 1 School of Life Sciences, Institute of Brain Science and State Key Laboratory of Medical Neurobiology, Fudan University, Shanghai 200433, PR China; 2 School of Life Science and Technology, Tongji University, Shanghai 200092, PR China; Dalhousie University, CANADA

## Abstract

Arachidonic acid (AA) and its metabolites are important second messengers for ion channel modulation. The effects of extracellular application of AA and its non-metabolized analogue on muscle rNa_V_1.4 Na^+^ current has been studied, but little is known about the effects of intracellular application of AA on this channel isoform. Here, we report that intracellular application of AA significantly augmented the rNa_V_1.4 current peak without modulating the steady-state activation and inactivation properties of the rNa_V_1.4 channel. These results differed from the effects of extracellular application of AA on rNa_V_1.4 current. The effects of intracellular AA were mimicked by prostaglandin E_2_ but not eicosatetraynoic acid (ETYA), the non-metabolized analogue of AA, and were eliminated by treatment with cyclooxygenase inhibitors, flufenamic acid, or indomethacin. AA/PGE_2_-induced activation of rNa_V_1.4 channels was mimicked by a cAMP analogue (db-cAMP) and eliminated by a PKA inhibitor, PKAi. Furthermore, inhibition of EP2 and EP4 (PGE_2_ receptors) with AH6809 and AH23848 reduced the intracellular AA/PGE_2_-induced increase of rNa_V_1.4 current. Two mutated channels, rNa_V_1.4S56A and rNa_V_1.4T21A, were designed to investigate the role of predicted phosphorylation sites in the AA/PGE_2_–mediated regulation of rNa_V_1.4 currents. In rNa_V_1.4S56A, the effects of intracellular db-cAMP, AA, and PGE_2_ were significantly reduced. The results of the present study suggest that intracellular AA augments rNa_V_1.4 current by PGE_2_/EP receptor-mediated activation of the cAMP/PKA pathway, and that the S56 residue on the channel protein is important for this process.

## Introduction

Arachidonic acid (AA) is a polyunsaturated fatty acid cleaved from cell membrane phospholipid molecules via the action of the enzyme PLA_2_. AA is a biologically active signaling molecule that plays important roles in neurons and muscle under both physiological and pathological conditions [1, 2]. Its effects include modulation of the activity of protein kinases, elevation of intracellular Ca^2+^ levels, and regulation of neuronal excitability [3–5]. AA and its metabolites have been shown to modulate ligand- and voltage-gated ion channels, such as NMDA and AMPA receptor channels, voltage-gated Na^+^ and K^+^ channels, and acid-sensing ion channels [6–9]. Regulation of ion channel activity by AA may occur via direct effects, where AA interacts directly with ion channel proteins, or through perturbation of the plasma membrane [7, 10, 11]. AA metabolites have been reported to indirectly modulate ion channels through oxygenases or cellular signal transduction pathways [12]. With the exception of our recent study in rat cerebellar granule neurons, most studies have focused on the effects of extracellularly-applied AA and have not investigated the effects of intracellular AA. Free intracellular AA serves as a key transient cell signaling intermediate and undergoes rapid enzymatic conversion to diverse metabolites, including prostaglandins (PGs, such as PGD_2_, PGE_2_ and thromboxane A_2_) and the leukotriene/lipoxin (LX) families of eicosanoids [13]. It would be interesting to compare the effects of extracellular versus intracellular AA application and to investigate the underlying mechanisms of cell response to intracellular application of AA.

Voltage-gated sodium channels (Na_V_) are one of the primary classes of ion channels responsible for driving cellular excitability in the nervous system and in skeletal and cardiac muscle. Na_V_ are important clinically because they play a central role in neuronal activity and in a number of disease pathologies [14]. The Na^+^ channel consists of one large α subunit, which creates a functional membrane channel, and small β subunits, which modulate the voltage-dependent Na^+^ channel [15]. To date, ten isoforms of the Na^+^ channel α subunit have been cloned and characterized (Nav1.1–1.9 and Nax). The majority of sodium currents in the brain neurons are mediated by Na_V_ 1.1–1.3 and Na_V_ 1.6 [16, 17], and it controls axonal action potential conduction and neurotransmitter release in presynaptic terminals [18]. Na_V_1.4 is the predominant voltage-gated Na^+^ channel isoform in skeletal muscle [19]. Mutations in the gene encoding Na_V_1.4 have been associated with non-dystrophic skeletal muscle pathologies, including paramyotonia congenita, hyperkalaemic periodic paralysis, and potassium-aggravated myotonia [20]. Therefore, understanding the mechanisms of regulation of Na_V_1.4 channel activity is of clinical importance.

Our previous studies indicated that AA activates or inhibits sodium channel current (*I*_Na_) which is mainly composed of the Na_V_1.2 α-subunit [21] in rat cerebellar granule cells (GCs) depending on the route of AA application, and that AA acts via either non-metabolic or metabolic pathways[22]. Extracellular AA directly inhibits *I*_Na_ and modifies Na_V_ channel kinetics, similar to results observed with the Na_V_1.4 channel [23]. In contrast, intracellular AA augments the *I*_Na_ current via metabolic pathways involving PGE_2_-mediated activation of cAMP/PKA pathways. However, the effect of intracellular AA on Na_V_1.4 channels is unclear. Moreover, the precise site phosphorylated by PGE_2_-mediated activation of cAMP/PKA pathways is lacked. Although Na_V_1.2 and Na_V_1.4 α subunit isoforms have greater than 60% amino acid sequence identity, these channels exhibit functional differences in gating, conductance and ion selectivity, which result in tissue-specific physiological functions and subtle differences in their pharmacological properties [24, 25]. Thus, further exploration of the effect of intracellular AA in muscle Na^+^ channels is required for a comprehensive understanding of the physiological role of AA.

In the present study, we used the whole-cell patch-clamp technique to investigate the effects of intracellular AA on the major α subunit of the Na^+^ channel protein complex from skeletal muscle (rNa_V_1.4). rNa_V_1.4 channels were transiently transfected into stable cultured human embryonic kidney cells (HEK 293), which lack voltage-dependent Na^+^ channels. Additionally, two site-directed mutants were designed to determine whether there is a phosphorylation site for PKA. Our studies indicate that intracellular AA activates rNa_V_1.4 currents via the AA→PGE_2_→EP receptor→cAMP/PKA pathway, and that the PKA phosphorylation site at S56 on the channel protein may be involved in the activity of AA.

## Materials and Methods

### Cell culture and rNa_V_1.4 transfection

HEK 293 cells were cultured in 1640 medium supplemented with 10% fetal calf serum in a 5% CO_2_ incubator at 37°C. Cells were trypsinized, diluted with culture medium, and grown in 35-mm dishes. The rat Na_V_1.4 sodium channel α subunit was cloned into pEGFP-N1 (Clontech Laboratories, Inc., Mountain View, CA, USA), as described previously [23]. Transient transfections were carried out using Lipofectamine^TM^ 2000 Transfection Reagent (Life Technologies Corporation, Carlsbad, CA, USA) when cells reached 40 to 60% confluence. The cells were used for electrophysiological recordings four to five days after transfection. Individual transfected cells were visualized based on their expression of green fluorescent protein.

### Patch clamp recordings

Whole-cell currents of HEK 293 cells were recorded using a conventional patch-clamp technique. Prior to current recordings, the culture medium was replaced with a bath solution containing (in mM): NaCl 145, KCl 2.5, HEPES 10, MgCl_2_ 1, and glucose 10 (pH adjusted to 7.4 using NaOH). Soft glass recording pipettes were filled with an internal solution containing (in mM): CsCl 142, NaCl 3, HEPES 10, MgCl_2_ 2, and EGTA 5 (pH adjusted to 7.3 using CsOH). The pipette resistance was 4–7 MΩ after being filling with the internal solution. All recordings were performed at room temperature (23-25C°).

### Data acquisition and analysis

All currents were recorded using an Axopatch 200B amplifier (Axon Instrument, Foster City, CA, USA) operated in voltage-clamp mode. The computer was connected to the recording equipment with a Digidata 1300 analog-to-digital (A/D) interface. Currents were digitally sampled at 100 μs (10 kHz) and were filtered with a 3-kHz, three-pole Bessel filter. Currents were corrected on-line for leak and residual capacitance transients by a P/4 protocol. Data acquisition and analysis were performed with pClamp 8.01 software (Axon Instruments, Union City, CA, USA) and/or Origin 6.1 (Origin Lab, Northampton, MA, USA). Values are given as mean ± S.E.M., with n representing the number of cells. Statistical analysis was performed using the Student’s t-test with paired comparisons where relevant. When multiple comparisons were made, data were analyzed by a one-way ANOVA test, followed by Tukey’s test when significant differences were observed.

### Reverse transcriptase-mediated PCR analysis

RNA was isolated from HEK 293 cells using a RNeasy Mini Kit (Qiagen). First-strand cDNA synthesis was performed on 3μg of total RNA with MMLV (Moloney murine leukaemia virus) reverse transcriptase and oligo (dT)18 according to the manufacturer’s instructions (Promega). A 2 μl aliquot of the total reverse transcription reaction volume (20μl) was used as the template in semi-quantitative reverse transcriptase-mediated PCR amplification, ensuring that the amount of amplified product remained in linear proportion to the initial template present in the reaction. A 5 μl aliquot of the PCR reaction was separated on a 1% agarose gel. For the negative control, PCR was performed using isolated RNA as the template. Primers used were 5'-ATCATGGTGGTGTCGTGCATC-3' and 5'-TGTGCTTAGAAGTGGCTGAGGC-3' for PTGER1 (NM_000955.2), 5'- CTGCTCCTTGCCTTTCACGAT-3' and 5'-AGCTGGATCTTCAATGCAGCC-3' for PTGER2 (NM_000956.3), 5'-TCCTCGTTGTTCATCGCCA-3' and 5'-TCTCCGTGTGTGTCTTGCAGTG-3' for PTGER3 (NM_001126044.1), and 5'-CCGAAGATTTGGCAGTTTCCA-3' and 5'-ACACTCATGGCGCAGATGATG-3' for PTGER4 (NM_000958.2). The PCR products were all approximately 500 bps in length.

### Prediction of phosphorylation sites and site-directed mutagenesis

The online program Scansite (http://scansite.mit.edu) was used to predict potential PKA phosphorylation sites in rNa_V_1.4. Optimal phosphorylation sites for particular protein Ser/Thr kinases were predicted using the matrix of selectivity values for amino acids at each position relative to the phosphorylation site as determined from the oriented peptide library technique. We evaluated the surface accessibility, score, and percentile parameters of all candidate PKA phosphorylation sites and chose two candidates, S56 and T21, for further study. We performed site-directed mutagenesis at the S56 and T21 sites using the Quikchange II-E Site-Directed Mutagenesis Kit (Agilent Technologies). The PCR primers used are as follows:

rNa_V_1.4S56A: 5'-GGAAGCCACGCGCTGACCTGGAAGC-3'

5'-GCTTCCAGGTCAGCGCGTGGCTTCC-3'

rNaV1.4T21A: 5'-TGCCTGCGCCCCTTCGCCCCAGAGTCCCTGGCA-3'

5'-TGCCAGGGACTCTGGGGCGAAGGGGCGCAGGCA-3'

### Chemicals

All drugs, with the exception of fetal calf serum, were purchased from Sigma-Aldrich (St. Louis, MO, USA). 1640 culture medium and antibiotic–antimycotic solution were obtained from Gibco Life Technologies (Grand Island, NY, USA). Arachidonic acid, 5,8,11,14-eicosatetraynoic acid, flufenamic acid, indomethacin, prostaglandin E_2_, AH6809 and AH23848 were first dissolved in DMSO and then diluted in extracellular or intracellular solution, with a final DMSO concentration <0.2%. At these low doses, DMSO alone does not modulate *I*_Na_.

## Results

### Intracellular AA increased rNa_V_1.4 current amplitude without modifying steady-state activation and inactivation properties of the channel

**Fig 1:**
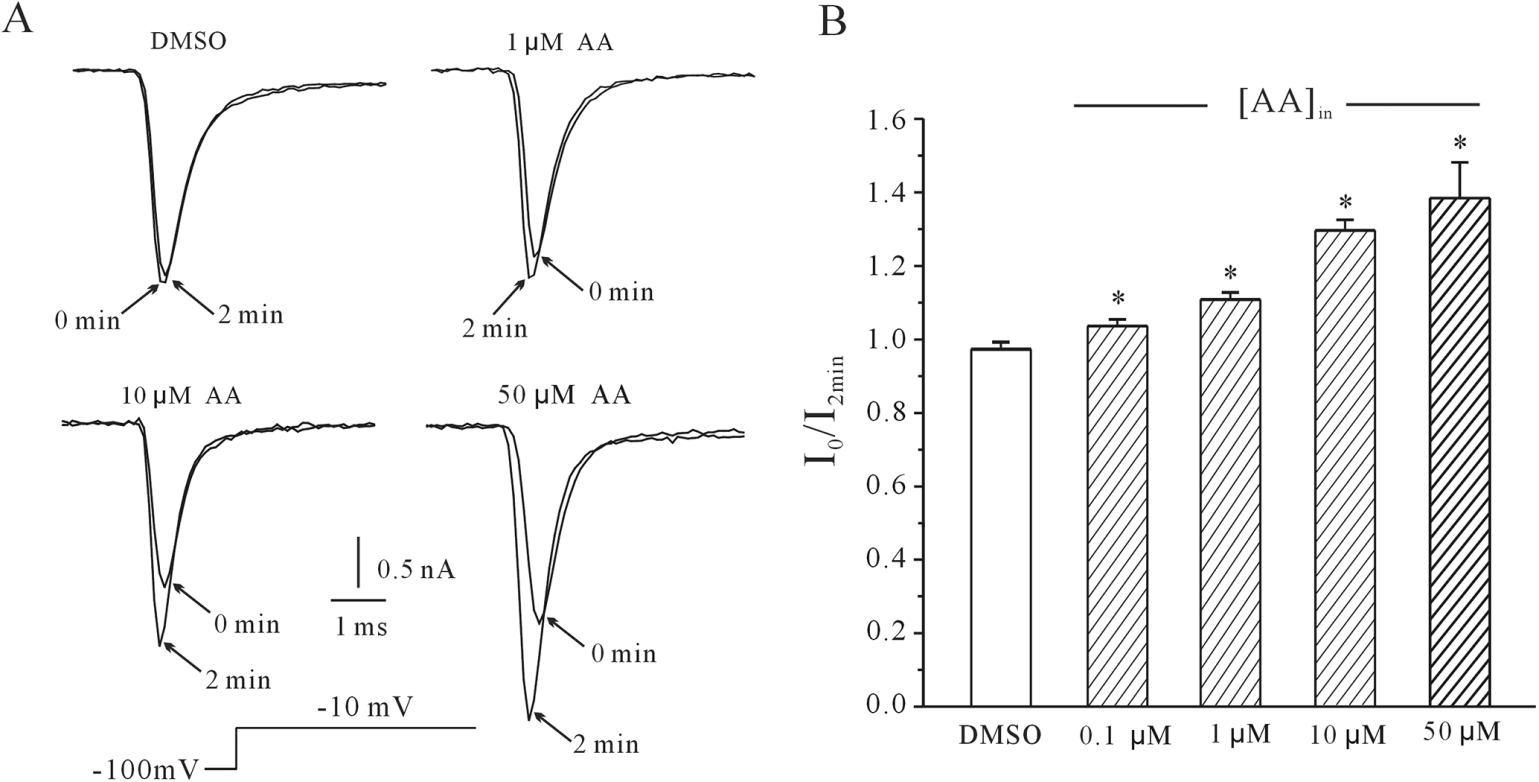
Concentration-dependent increase of rNav1.4 current in response to intracellular application of AA. (A) Superimposed rNa_V_1.4 currents evoked by a 10 ms depolarizing pulse from a holding potential of -100 to -10 mV. Current traces were obtained in the absence and presence of intracellular AA at concentrations of 1 μM, 10 μM, and 50 μM. An intracellular solution containing 0.2% DMSO was used as a control and did not affect the rNa_V_1.4 current. (B) The activating effect of different AA concentrations (0.1 μM, 1 μM, 10 μM, and 50 μM) on rNa_V_1.4 current. *P<0.05 compared to control, using a one-way ANOVA test.

The effect of intracellular AA on rNa_V_1.4 current amplitude was first investigated. The rNa_V_1.4 current evoked by a depolarization step to –10 mV from a holding potential of -100 mV for evoking the large current amplitude. When 10 μM AA was delivered intracellularly via pipette, the rNa_V_1.4 current evoked increased with time after the establishment of the whole-cell configuration. Moreover, the AA-induced increase of the rNa_V_1.4 current was concentration-dependent (Fig 1A). The rNa_V_1.4 current amplitude recorded 2 minutes after the establishment of the whole-cell configuration increased by 5.27 ± 1.35% (*n* = 5), 11.72 ± 1.04% (*n* = 6), 30.72 ± 1.83% (*n* = 10) and 42.37 ± 6.49% (*n* = 4) with intracellular AA at concentrations of 0.1 μM, 1 μM, 10 μM, and 50 μM, respectively (*P*<0.05 by the one-way ANOVA test). To rule out the possibility that DMSO might contribute to the rNa_V_1.4 current response, a control solution containing 0.2% DMSO was applied and the current amplitude lightly decrease 1.275 ± 1.015%, which was not a significant difference comparing with control (*n* = 5, *P* > 0.05). Statistical analysis of these data is shown in Fig 1B.

**Fig 2:**
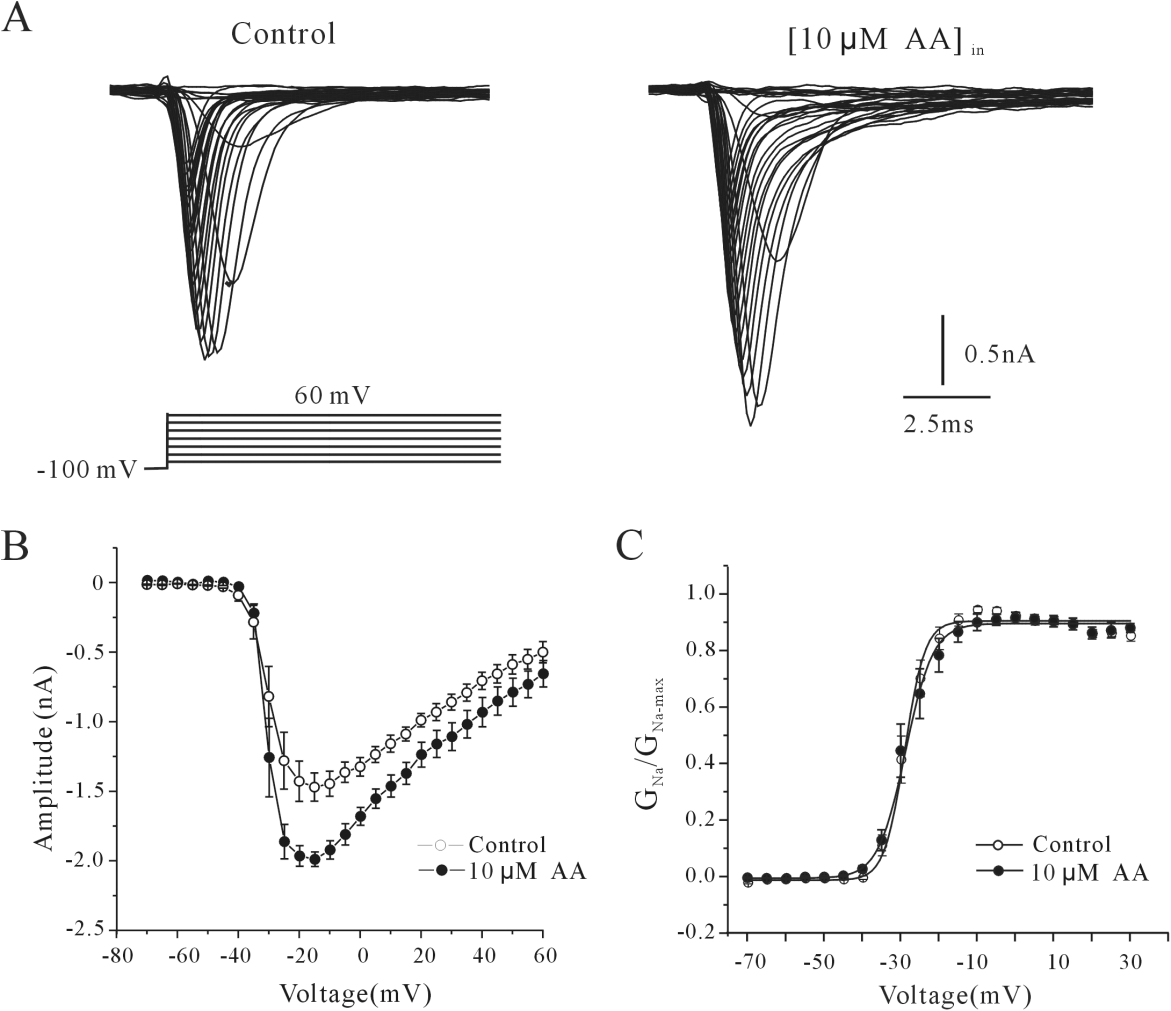
Effect of AA on the steady-state activation of rNa_V_1.4 currents_._ (A) Effect of AA on steady-state activation of rNa_V_1.4 currents. Cells were held at -100 mV and depolarized in 10 mV steps from -70 to +60 mV at intervals of 10 s. (B) Voltage-dependent activation curves of rNa_V_1.4 currents in the absence and presence of 10 μM AA. (C) Normalized conductance as a function of the command potential in the absence or presence of 10 μM AA. Conductance was obtained by *g*_Na_ = *I*_Na_ /(*V*_m1/2_–*V*_rev_). Data points were fitted with the Boltzmann function *g*_Na_/*g*_Na-max_ = 1/{1+exp[(*V*_m1/2_–*V*_m_)/*k*]}. Data were obtained from seven cells and are expressed as mean ± S.E.M

The effect of intracellular AA on the steady-state activation properties of the rNa_V_1.4 channel was then studied using appropriate voltage protocols. rNa_V_1.4 currents were evoked by 20 msec depolarizing pulses from a holding potential of -100 mV to potentials between -70 mV to +60 mV in steps of 5 mV at intervals of 10 sec (Fig 2A). The voltage-current curve presented in Fig 2B shows that the maximum activation potential changed from -17.14 ± 3.60 mV to -15.71 ± 3.69 mV in the presence of 10 μM intracellular AA (*P*>0.05, *n* = 7 for control and *n* = 7 for AA). We obtained steady-state activation of rNa_V_1.4 by normalizing the conductance as a function of the command potential. The data points were calculated using the equation *g*_Na_ = *I*_Na_ / (*V*_m_–*V*_rev_), where *g*_Na_ is the membrane Na^+^ conductance, *V*_m_ is the membrane potential, and *V*_rev_ is the reversal potential for Na^+^. For both the control and AA treatments, the ratios were fitted to a Boltzmann function in the form of *g*_Na_/*g*_Na-max_ = 1/ {1+exp [(*V*_m1/2_–*V*_m_)/*k*]}, and the half-activation potential was obtained (Fig 2C). According to these curves, the currents were half-activated at -32.43 ± 1.11 mV and -30.08 ± 1.02 mV (n = 26 for control and 24 for AA, *P>*0.05), the slope factor was 3.0 ± 0.48mV and 3.32 ± 0.33 mV (*P>*0.05) in the absence and presence of 10 μM AA, suggesting that intracellular AA did not significantly shift the voltage-dependence property of the steady-state activation of rNa_V_1.4.

**Fig 3:**
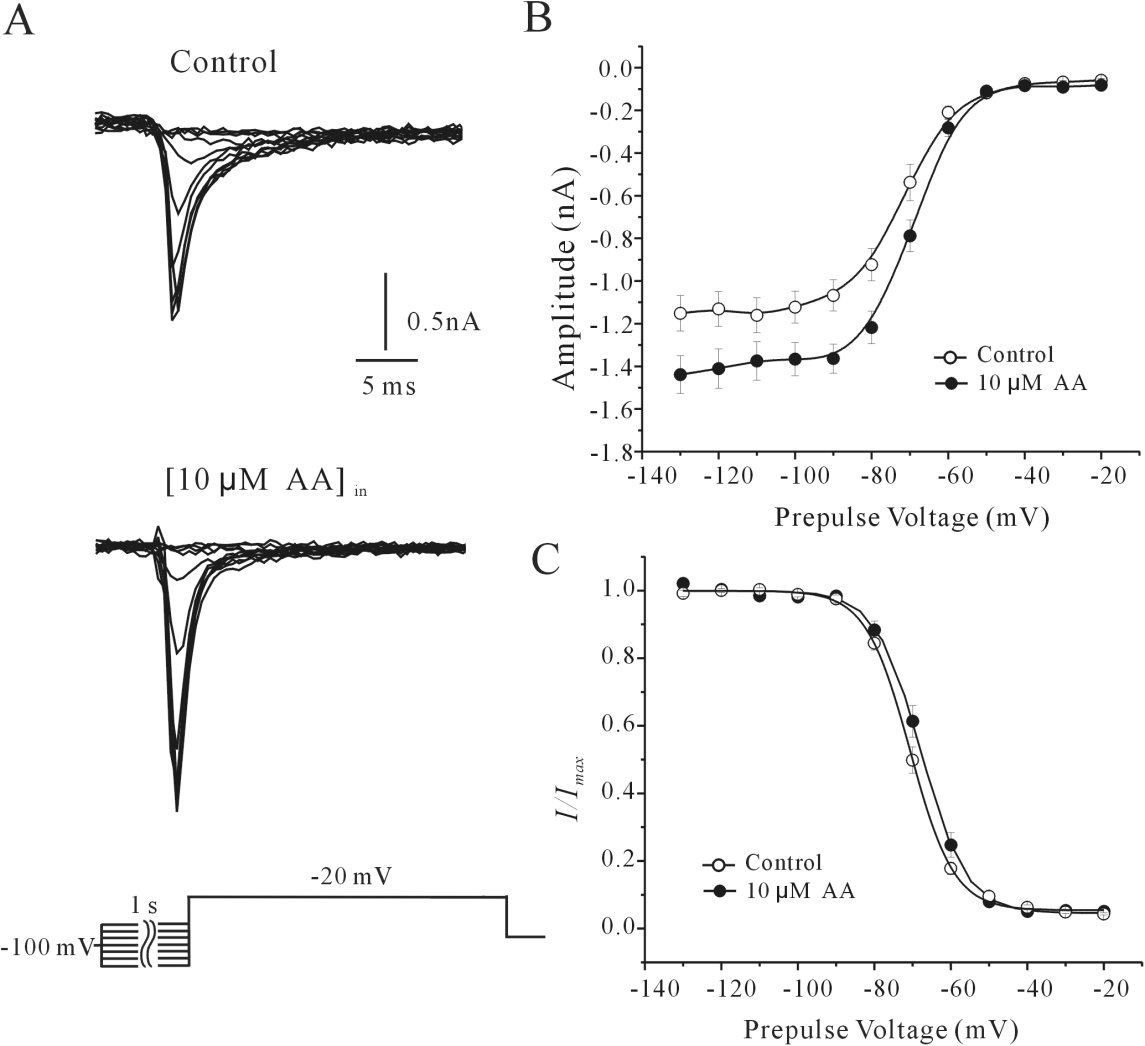
Effect of AA on the steady-state inactivation of rNa_V_1.4 currents. (A) Control currents (top) and currents following the application of 10 μM AA (bottom). Conditioning pre-pulses of 1s from -130 to -10 mV in increments of 10 mV were applied before the test pulse to -20 mV. The voltage protocol is shown below the current recordings. (B) Steady-state inactivation curves of rNa_V_1.4 currents in the absence and presence of 10 μM AA. The abscissa indicates the conditioning pre-pulse potentials. (C) Peak current amplitude normalized to the maximum current plotted against the pre-pulse potential. Normalized current points were fitted with the Boltzmann function *I*_Na_/*I*_Na-max_ = 1/{1+exp[(*V*_m_–*V*_m1/2_)/*k*]}+*A*. Data were obtained from twenty-five cells and are expressed as mean ± S.E.M.

We also studied the effect of AA on the voltage dependence of the steady-state inactivation of rNa_V_1.4. Current was elicited using 1s conditioning pre-pulses ranging from -130 mV to -10 mV in steps of 10 mV prior to a -20 mV test pulse. Fig 3A shows the currents obtained from cell recordings with normal intracellular solution (Control) or with 10 μM AA in the intracellular solution (AA). A curve illustrating the relationship between the rNa_V_1.4 current peak and the pre-pulse potential is shown in Fig 3B. The steady-state inactivation curve was fitted using the Boltzmann equation *I*_rNav1.4_ /*I*_rNav1.4max_ = 1/ {1+exp [(*V*_m_–*V*_m1/2_)/*k*]} +*A*. In the 25 cells studied (*n* = 15 for control and *n* = 10 for AA), half-inactivated potential was -70.72 ± 0.86 mV and -67.91 ± 1.11 mV, slope factor was 4.36 ± 0.42 and 5.90 ±0.25 in the absence and presence of AA, respectively (*P*>0.05). Therefore, intracellular AA did not significantly shift the steady-state inactivation curve.

**Fig 4:**
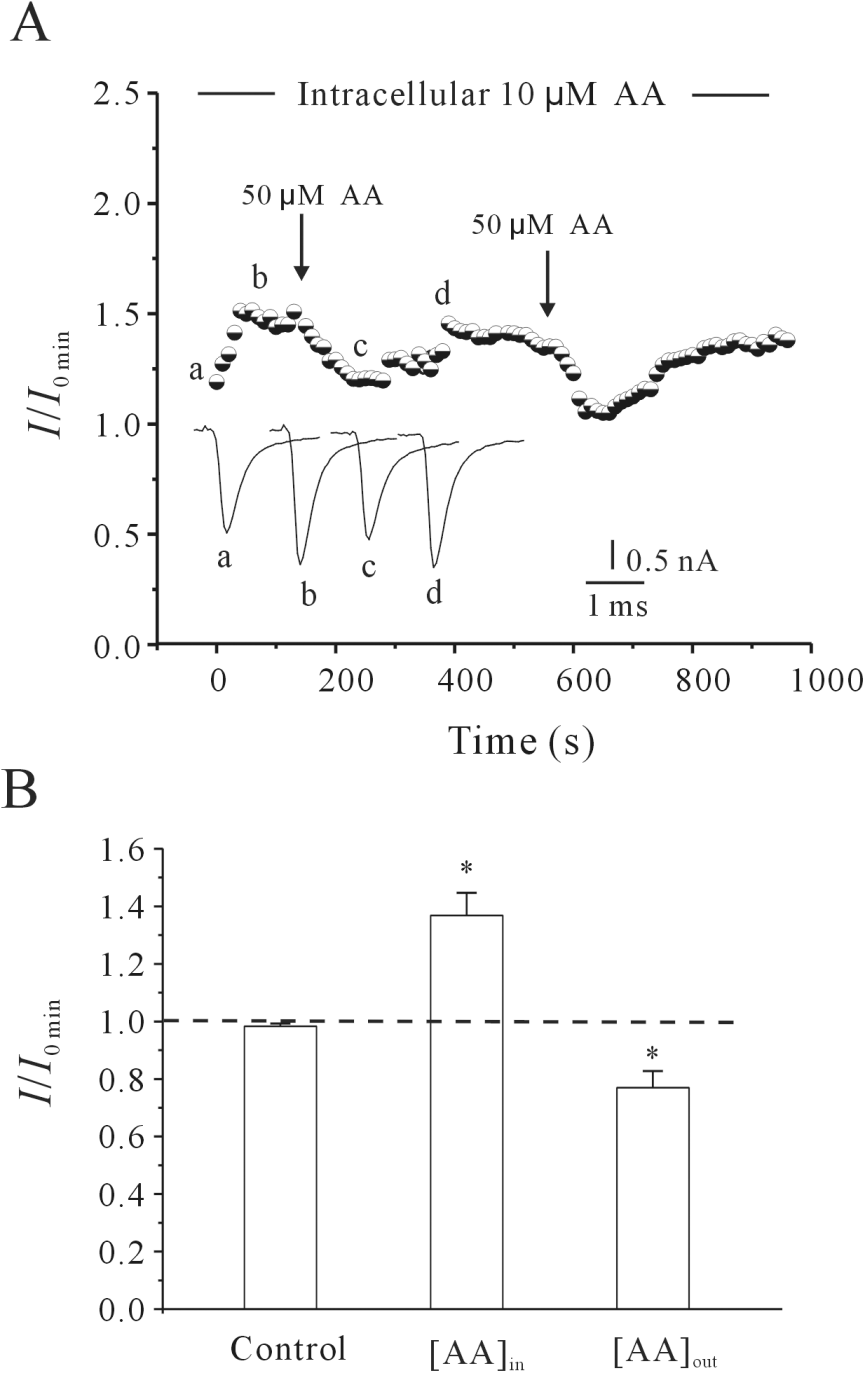
Effect of extracellular AA on the rNa_V_1.4 current after intracellular application of AA. (A) Representative sample showing the time course of rNa_V_1.4 current amplitude under control conditions, after intracellular application of 10 μM AA, and after extracellular application of 50 μM AA. The letters (a, b, c and d) noted on the curves correspond to the superimposed rNa_V_1.4 current traces in the insets. *I*_0_ denotes the current obtained at membrane rupture. (B) Statistical analysis of the effect of internal AA ([AA]_in_) and external AA ([AA]_out_) on the amplitude of rNa_V_1.4 current. *, P<0.01 compared to control, using the Student’s t test.

Our previous study revealed that extracellular AA had an inhibitory effect on the rNa_V_1.4 current[23]. We thus tested whether intracellular application of AA could affect the inhibitory effect induced by extracellular AA. Fig 4A illustrates a representative experiment in which external AA was applied twice to the same cell while 10 μM AA was added to the pipette solution. The current amplitude increased over time with intracellular AA. After a stable rNa_V_1.4 current had been obtained, external application of AA (50 xM) still caused a reversible decrease in the current amplitude by 23.05 ± 5.75% (*P*<0.01, *n* = 5), which was similar with external AA alone [23]. Statistical analysis of these data is shown in Fig 4B.

### Intracellular AA increases the rNa_V_1.4 current via its metabolic product, PGE_2_, and via the EP receptor

**Fig 5:**
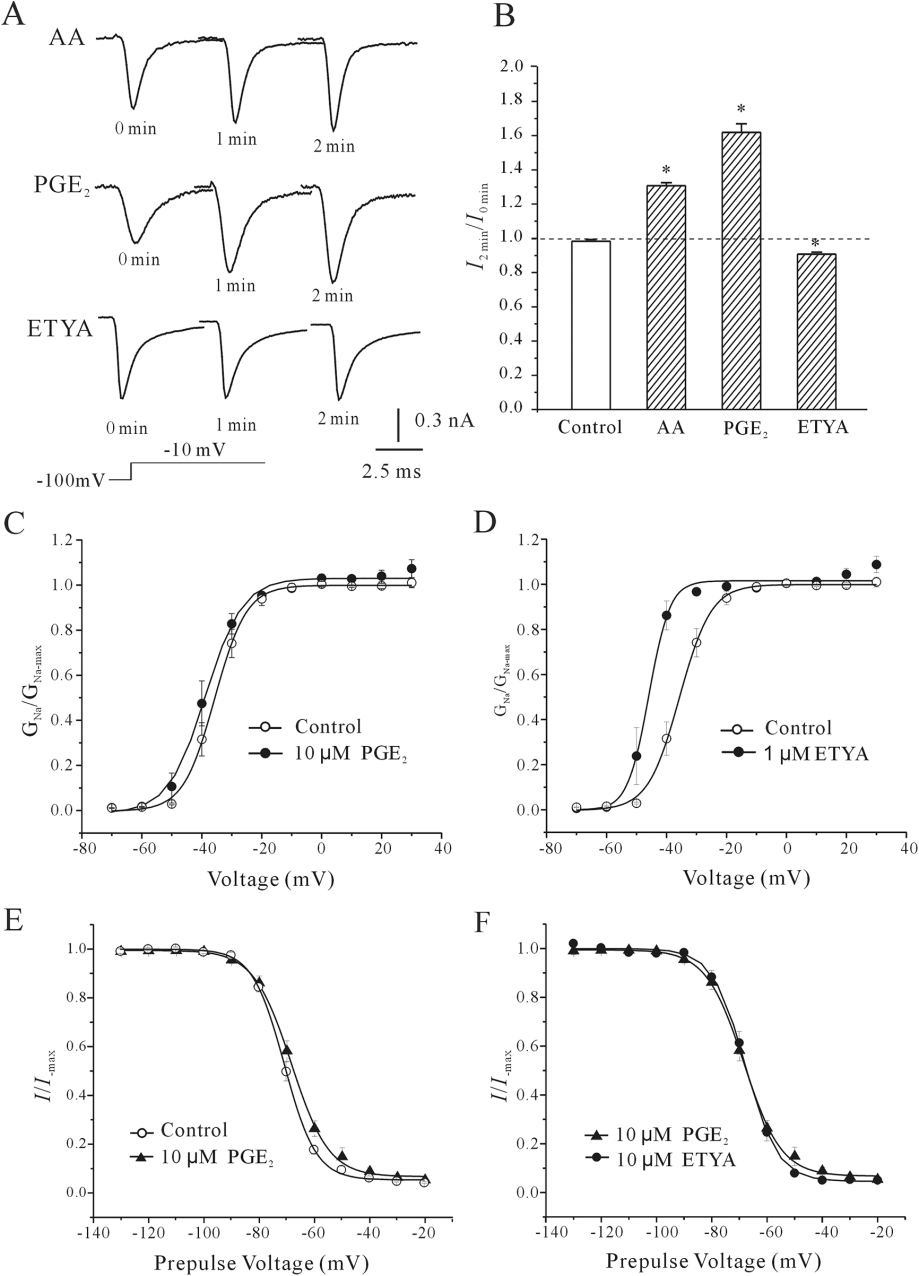
Comparison of the effects of intracellular application of AA, PGE_2_, and ETYA on rNaV1.4 current amplitude and gating kinetics. (A) Representative sample showing the time course of rNa_V_1.4 current amplitude after intracellular application of 10 μM AA, 10 μM PGE_2_, or 1 μM ETYA. rNa_V_1.4 current traces were taken from the initial control levels (at membrane rupture, 0 min), and after intracellular application of AA, PGE_2_, or ETYA for 1 min and 2 min. (B) Statistical analysis of the effect of intracellular AA, PGE_2_, and ETYA on the amplitude of the rNa_V_1.4 current. Results represent the mean ± S.E.M. obtained from nine or ten cells. *, P<0.05 compared to control, using the Student’s t test. (A-B) Steady-state activation curve of rNa_V_1.4 currents in the absence and presence of intracellular PGE_2_ or ETYA. (C-D) Comparison of normalized conductance as a function of the command potential in the absence or presence of PGE_2_. The abscissa indicates the conditioning pre-pulse potentials. Data were obtained from twenty-five cells and are expressed as mean ± S.E.M.

AA is known to be consumed through its conversion to prostaglandins and other products by the cyclooxygenase, lipoxygenase, and monooxygenase pathways[13]. Since the COX-2 metabolite prostaglandin E_2_ (PGE_2_) is involved in the intracellular AA-induced modulation of neuronal Na^+^ current, we replaced intracellular AA with PGE_2_ or eicosatetraynoic acid (ETYA), the non-metabolized analogue of AA to determine whether it is AA itself or its metabolic products that directly modulate(s) the rNa_V_1.4 current (Fig 5A and 5B). Application of 10 μM PGE_2_ in the pipette solution induced a similar effect as AA on the rNa_V_1.4 current. The current amplitude increased by approximately 61.94 ± 4.96% (n = 10, *P<*0.01). In contrast, application of 1 μM ETYA to the intracellular solution gradually lowered the rNa_V_1.4 current by approximately 9.30 ± 1.18%. The normal intracellular solution alone did not affect rNa_V_1.4 current during the two-minute recording. Statistical analysis of these data is shown in Fig 5B.

To further address the role of PGE_2_ in the intracellular AA-induced increase of the rNa_V_1.4 current, the effects of PGE_2_ and ETYA on the steady-state activation and inactivation properties of the rNa_V_1.4 channel were compared to that of AA using the same voltage protocols as above (Fig 2A and Fig 3A). The half-activation potential was -39.56 ± 2.11 mV in the presence of 10 μM PGE_2_ (n = 14 for ctrl, n = 10 for PGE_2_), similar to the -34.98 ± 1.62 mV half-activation potential obtained in the presence of intracellular AA (*P*>0.05). In contrast, the half-activation potential was -45.41 ± 1.81 mV in the presence of 1 μM ETYA (*P<*0.05, n = 8). There was a significant difference between the effects of ETYA and intracellular AA on the voltage-dependence of the steady-state activation of rNa_V_1.4 (Fig 5C and 5D). However, there was no significant shift in the steady-state inactivation curve in the present of intracellular 10 μM PGE_2_ or 1 μM ETYA (Fig 5E and 5F). Statistical analysis of these data is shown in Table 1 and Table 2.

**Table 1:** The half-activation potential and slope factor of I-V curve of rNaV1.4.

Intracellular buffer	n	half-activation potential (mV)	slope factor (mV)
normal	26	-32.43±1.11	3.00±0.48
10μM AA	24	-30.08±1.02	3.32±0.33
10μM PGE_2_	10	-39.56±2.11 *	6.17±0.59
1μM ETYA	15	-45.41±1.81 *	3.49±0.44

Data are shown as mean ± S.E.M., Significance was tested using Oneway ANOVA test.

*, P<0.01 compared with control.

**Table 2:** The half-inactivation potential and slope factor of inactivation curve of rNaV1.4.

Intracellular buffer	n	half-inactivation potential (mV)	slope factor (mV)
normal	15	-70.72±0.86	4.36±0.42
10μM AA	10	-67.91±1.11	5.90±0.25
10μM PGE_2_	10	-68.14±1.05	6.78±0.28
1μM ETYA	15	-69.44±2.14	6.96±0.26

Data are shown as mean ± S.E.M., Significance was tested using Oneway ANOVA test.

**Fig 6:**
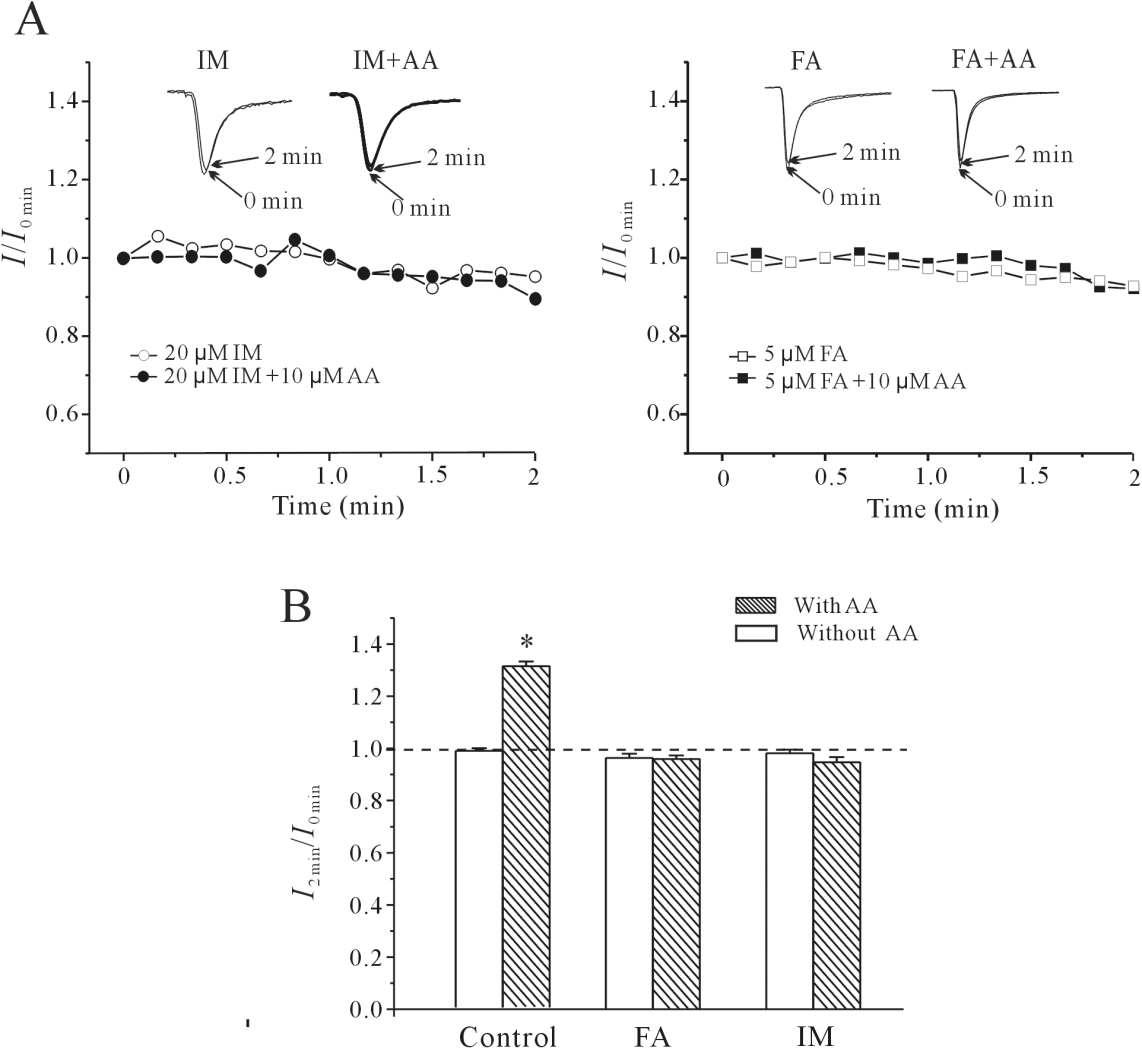
The effect of COX inhibition on rNa_V_1.4 current and AA-induced increases in rNa_V_1.4 current. (A) Time course of rNa_V_1.4 current amplitude obtained after intracellular application of 20 μM indomethacin (IM) or 5 μM flufenamic acid (FA), in the presence or absence of AA. The insets show rNa_V_1.4 current traces taken from the same cells. (B) Statistical analysis of the effect of indomethacin and flufenamic acid on the amplitude of the rNa_V_1.4 current and on the AA-induced increase in rNa_V_1.4 current. Results represent the mean ± S.E.M. *, P<0.05 compared to the corresponding control, using the Student’s t test.

Next, flufenamic acid and indomethacin, two cyclooxygenase inhibitors, were used to confirm that AA metabolites mediate the effect of intracellular AA on the rNa_V_1.4 current. Application of 5 μM flufenamic acid or 20 μM indomethacin alone in the bath solution did not significantly modify the rNa_V_1.4 current amplitude. Within two minutes after the establishment of the whole cell configuration, the current amplitude was only reduced by 3.70 ± 1.66% (*n* = 8, *P*>0.05) and 1.83 ± 1.34% (*n* = 8, *P*>0.05), respectively. Adding AA to the pipette solution together with flufenamic acid or indomethacin eliminated the AA-induced increase of the rNa_V_1.4 current (Fig 6A and 6B). When AA was applied intracellularly, the current amplitude was reduced by 4.06 ± 1.37% (n = 15, *P*>0.05) and 5.32 ± 1.97% (n = 7, *P*>0.05) in the presence of flufenamic acid or indomethacin, respectively.

**Fig 7:**
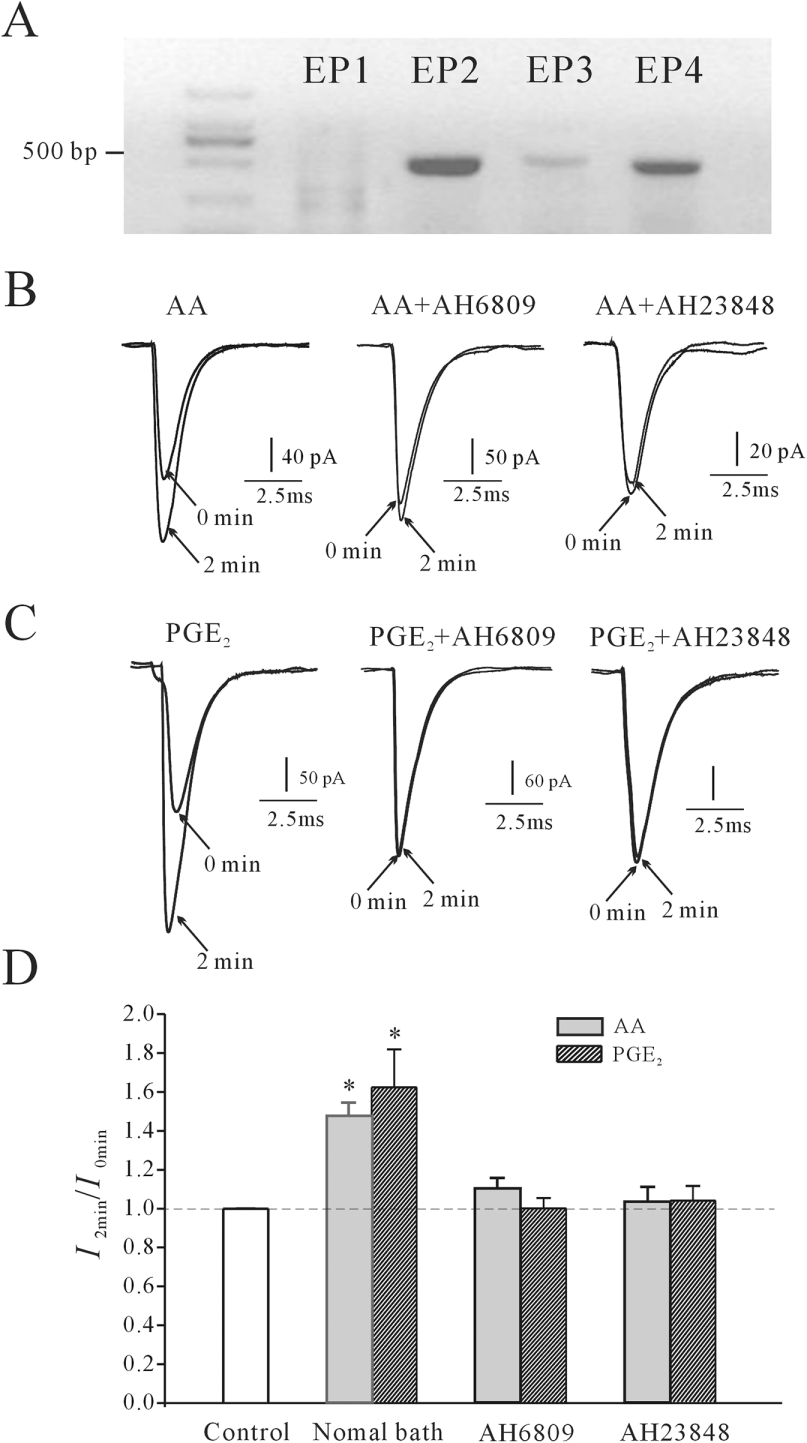
The effects of EP2/EP4 receptor antagonists on AA/PGE_2_-induced increases in rNa_V_1.4 current. (A) RT-PCR analysis shows the presence of EP receptor mRNA in HEK 293 cells. (B) rNa_V_1.4 current traces taken at the initial time point (0 min, at membrane rupture) and after intracellular application of AA in the absence or presence of 25 μM AH6809 or AH23848 for 2 min. (C) rNa_V_1.4 current traces taken at the initial time point (0 min, at membrane rupture) and after internal infusion of PGE_2_ in the absence or presence of 25 μM AH6809 or AH23848 for 2 min. (E) Statistical analysis of the effects of AH6809 or AH23848 on AA/PGE_2_-induced increases in rNa_V_1.4 current. Results are mean ± S.E.M. from four to eight cells. *, P<0.05, compared to control (without AA or PGE_2_), using the Student’s t test.

We then investigated whether PGE_2_ receptors were associated with the AA/PGE_2_-induced increase of the rNa_V_1.4 current. Reverse transcriptase-mediated PCR analysis was first used to detect the presence of EP receptor mRNA in HEK 293 cells. PCR results showed that EP2 and EP4 are the major EP receptors expressed in HEK 293 cells, while EP3 mRNA is less abundant (Fig 7A). Thus, EP2 and EP4 receptor antagonists AH6809 and AH23848 were used for further exploration. Bath infusion of AH6809 or AH23848 significantly inhibited the effects of AA or PGE_2_ on the rNa_V_1.4 current (Fig 7B and 7C). In the presence of AH6809 or AH23848, the AA-induced increase of the rNa_V_1.4 current was significantly reduced to 9.7±4.35% (n = 8, *P*<0.05) and 3.61±6.45% (n = 7, *P*<0.05), respectively. Similarly, the PGE_2_-induced increase in the rNa_V_1.4 current was significantly reduced to 4.28±8.42% (n = 6, *P*<0.05) and 1.68±4.48% (n = 7, *P*<0.05), respectively. Statistical analysis of these results is shown in Fig 7D. These results indicate that EP2 and EP4 receptors are associated with AA/PGE_2_-induced increase of the rNa_V_1.4 current.

### cAMP/PKA pathways and rNa_V_1.4 S56 phosphorylation is involved in AA/PGE2-induced increase of the rNa_V_1.4 current

**Fig 8:**
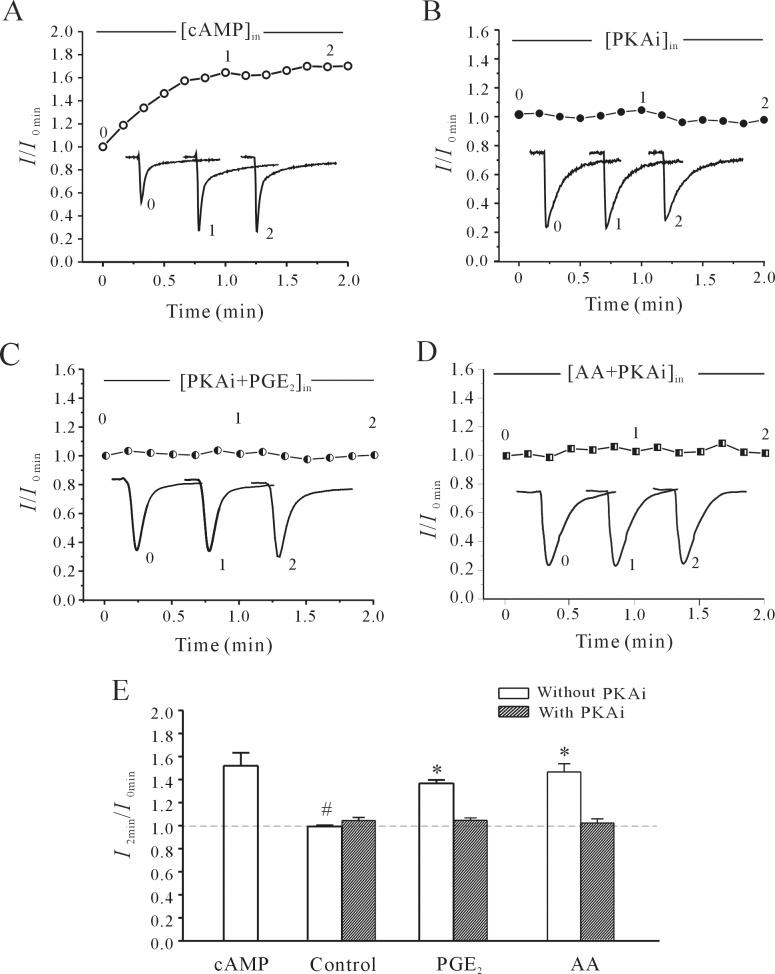
Effect of activating or blocking cAMP/PKA pathways on AA/PGE_2_-induced increases in rNa_V_1.4 current. (A) Time course of changes in rNa_V_1.4 current amplitudes induced by intracellular application of 10 μM db-cAMP. (B) Time course of changes in rNa_V_1.4 current amplitudes induced by 50 nM PKAi. (C and D) Time course of changes in rNa_V_1.4 current amplitudes induced by 10 μM PGE_2_ or 10 μM AA in the presence of PKAi. The time points (0 min, 1 min and 2 min) noted on the curves correspond to the superimposed rNa_V_1.4 current traces in the insets. (E) Statistical analysis of the effects of cAMP or PKAi alone on the rNa_V_1.4 current, and the effect of PKAi on PGE_2_-induced increases in rNa_V_1.4 current. Results are mean ± S.E.M. from four to eight cells. *, P<0.05 compared to PKAi group; #, P<0.05 compared to intracellular application of cAMP, using the Student’s t test.

The EP2/EP4 receptor is known to activate PKA pathways. To investigate whether the PKA pathway is involved in AA/PGE_2_-induced increase of the rNa_V_1.4 current, the effects of PGE_2_ and AA on the rNa_V_1.4 current were investigated in the presence of a PKA activator or inhibitor. Intracellular application of 10 μM db-cAMP (dibutyryl cAMP), a membrane permeable cAMP analogue, mimicked the AA- or PGE_2_-induced effect on rNa_V_1.4 (Fig 8A) and significantly increased the rNa_V_1.4 current amplitude by 51.4±11.3% (n = 7, *P*<0.05). Furthermore, the AA- or PGE_2_-induced increase in rNa_V_1.4 current amplitude was attenuated by applying 50 nM PKAi, a bioactive peptide fragment 6–22 amide which inhibited PKA activity (Fig 8C and 8D). In the presence of 50 nM PKAi, the PGE_2_-induced increase in rNa_V_1.4 current was significantly reduced from 61.94 ± 4.96% (n = 10) to 24.2% (n = 8, *P*<0.05). Similarly, application of 50 nM PKAi reduced the AA-induced increase of rNa_V_1.4 current from 47.443±7.12% (n = 13) to 1.82±3.52% (n = 11, *P*<0.05). Intracellular application of 50 nM PKAi alone did not affect the rNa_V_1.4 current amplitude (Fig 8B), Statistical analysis of these results is shown in Fig 8E. These results suggest that the cAMP/PKA pathway is associated with the AA/PGE_2_-induced increase of the rNa_V_1.4 current.

Skeletal muscle sodium channels are phosphorylated by PKA despite lacking the large loop I-II that is the target of PKA in other sodium channels [26]. To detect potential PKA phosphorylation sites on rNav1.4, we performed a Scansite search that revealed four potential PKA sites with low percentile scores: T21, S56, S251, and S899. Both S251 and S899 are located in the hydrophobic transmembrane region of the channel protein, a region that is difficult to phosphorylate. Therefore, we chose to investigate the other two sites, S56 and T21. We designed mutated rNav1.4 channel proteins with single-point mutations at these sites and investigated the effects of AA/PGE_2_ on these mutated channels.

**Fig 9:**
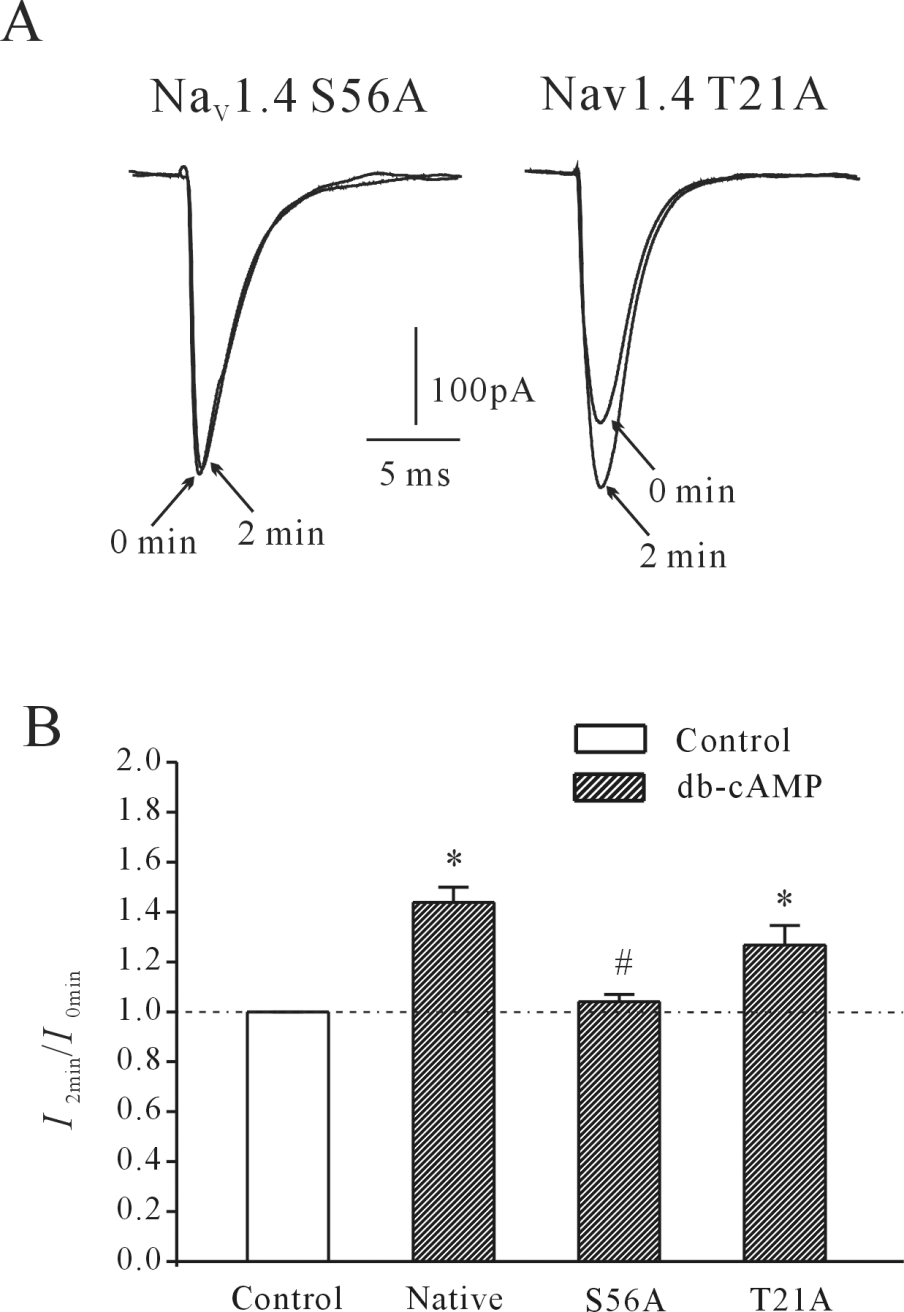
The effects of cAMP on mutant rNa_V_1.4T21A and rNa_V_1.4S56A currents. (A) Current traces taken at the initial time point (0 min, at membrane rupture) and after intracellular application of cAMP for 2 min in mutant rNa_V_1.4T21A and rNa_V_1.4S56A channels. (C) Statistical analysis of the effects of internal cAMP on currents in rNa_V_1.4S56A, rNa_V_1.4T21A, and wildtype rNa_V_1.4 channels. Results are mean ± S.E.M. from four to six cells. *, P<0.05, compared to control (without cAMP); #, *P*<0.05, compared to wildtype rNa_V_1.4 with intracellular application of cAMP.

**Fig 10:**
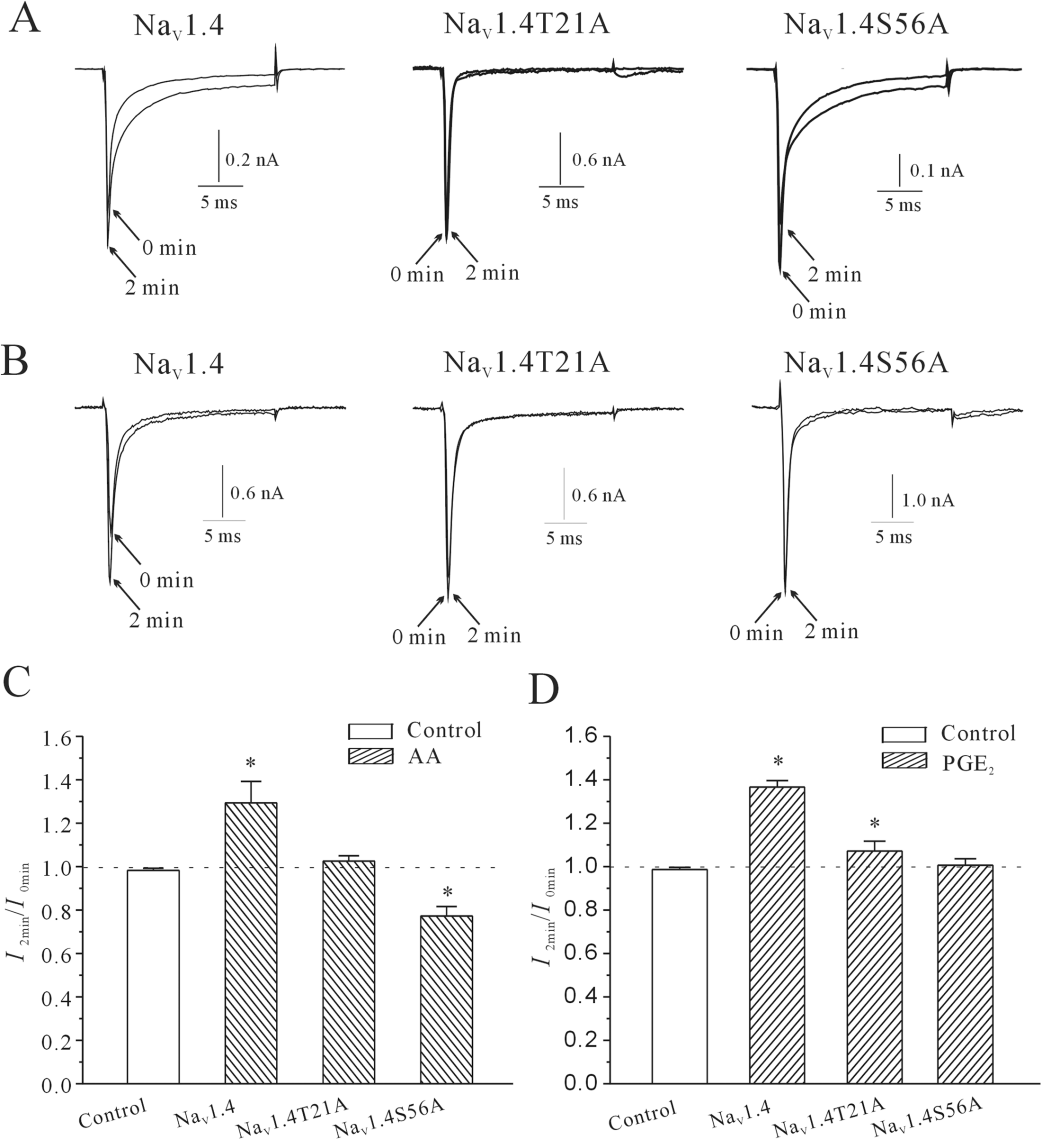
The effects of AA/PGE_2_ on currents in sodium channel mutants rNa_V_1.4T21A and rNa_V_1.4S56A. (A) Current traces taken at the initial time point (0 min, at membrane rupture) and after intracellular application of 10 μM AA for 2 min in wildtype rNa_V_1.4 and mutants rNa_V_1.4T21A and rNa_V_1.4S56A. (B) Current traces taken at the initial time point and after intracellular application of 10 μM PGE_2_ for 2 min in wildtype rNa_V_1.4 and mutants rNa_V_1.4T21A and rNa_V_1.4S56A. (C) Statistical analysis of the effects of internal AA and PGE_2_ on currents in wildtype rNa_V_1.4 channels and mutant rNa_V_1.4T21A and rNa_V_1.4S56A channels. Results are mean ± S.E.M. from four to six cells. *, P<0.05, compared to control, using the Student’s t test.

The two site-directed mutants, rNa_V_1.4S56A and rNa_V_1.4T21A, were designed to have their S56 serine or T21 threonine residue replaced with an uncharged alanine residue. Fig 9A and 9B show that intracellular 10 μM db-cAMP significantly reduced the rNa_V_1.4S56A current and rNa_V_1.4T21A current to 4.06±2.67% (n = 18, *P*>0.05) and 23.65±7.72% (n = 13, *P*<0.05), respectively. Moreover, the effect of intracellular AA and PGE_2_ on rNa_V_1.4T21A and rNa_V_1.4S56A was significantly diminished (Fig 10A and 10B). When 10 μM AA was delivered intracellularly via pipette, the rNa_V_1.4T21A current amplitude was only increased by 2.6±2.4% (n = 6). Furthermore, application of 10 μM AA only slightly decreased the current amplitude of rNa_V_1.4S56A by 22.7±4.3% (n = 4, *P*<0.05, compared with the effect of 10 μM AA on normal rNa_V_1.4 with the Student’s t test). This result was significantly different than the effect of intracellular application of 10 μM AA on normal rNa_V_1.4 current. Similarly, intracellular application of 10 μM PGE_2_ only increased the rNa_V_1.4T21A current and rNa_V_1.4S56A current by 7.2±4.5% (n = 4) and 2.8±1.5% (n = 11), respectively. These results are significantly different than the effect of intracellular application of 10 μM PGE_2_ on normal rNa_V_1.4 current (P<0.05, compared with the effect of 10 μM PGE_2_ on normal rNa_V_1.4 with the Student’s *t*-test). Statistical analysis of these results is shown in Fig 10C and 10D.

## Discussion

We previously reported that extracellular AA directly inhibited rNa_V_1.4 current due to the activity of the AA molecule itself [23]. Here, our results indicate that intracellular AA increased the rNa_V_1.4 current via metabolic pathways involving EP receptor-mediated activation of cAMP/PKA, and that the PKA phosphorylation site S56 on the rNa_V_1.4 channel protein may play a pivotal role in this regulation. These observations were largely consistent with our previous data in neuronal *I*_Na_ channels, which are mainly composed of the Na_V_1.2 α subunit [22]. However, we observed that AA significantly modified the maximum activation potential and the steady-state activation of neuronal *I*_Na_, but not that of Na_V_1.4, suggesting an isoform-specific effect. We revealed the pivotal role of the amino acid S56 in establishing the specific channel properties of Na_V_1.4. We also demonstrated that PKA phosphorylation is mechanistically involved in the modulation of rNa_V_1.4 by intracellular AA.

AA is known to directly inhibit several ion channels, including neuronal Na^+^ and skeletal Na^+^ channels, as we have previously reported [7, 10, 27–30]. This inhibitory effect is regarded as a consequence of the direct action of the AA molecule itself because 1) AA was applied extracellularly and 2) the inhibitory effects could be mimicked by EYTA, an AA analogue that cannot be metabolized to generate biologically active products. In this study, we delivered AA into the intracellular space by pipette solution and observed an augmented rNa_V_1.4 current. This phenomenon is in contrast to the effects of extracellular AA [23]. The augmented current induced by intracellular AA could be mimicked by PGE_2_, a metabolite of AA produced by cyclooxygenase activity, but not by EYTA. Moreover, the increased rNa_V_1.4 current could be eliminated by blocking AA conversion to PGE_2_ via cyclooxygenase inhibition. In addition, while rNa_V_1.4 currents were increased by intracellular AA, extracellular application of AA reduced rNa_V_1.4 current amplitudes, suggesting that intracellular and extracellular application of AA act through different mechanisms to elicit different effects on rNa_V_1.4 currents. Taken together, our data indicate that intracellular application of AA increases rNa_V_1.4 current via its metabolic product, PGE_2_, as was previously reported for neuronal *I*_Na_ [22]. These results further confirm that regulation of sodium channels by excessive intracellular AA occurs through the action of PGE_2_, a metabolite of AA, and that this regulation is a universal phenomenon, having been observed in both nerve tissue and skeletal muscle.

PGE_2_ is known to act through the G-protein coupled receptors EP1-4. Among the four EP receptors, only EP2 and EP4 active adenylate cyclase and induce cAMP elevation. EP3 activation can result in the inhibition or (rarely) elevation of cAMP levels, and EP1 is coupled to calcium mobilization [31]. We observed that HEK 293 cells mainly express three types of PGE_2_ receptors. The increase in rNa_V_1.4 current induced by intracellular AA/PGE_2_ could be mimicked by intracellular cAMP, but not PKC activation or Ca^2+^ release (data not shown), so we conclude that the mechanism of the PGE_2_ effect involves EP2 and EP4 receptors. EP2/EP4 antagonists significantly eliminated the increased rNav1.4 current induced by intracellular AA/PGE_2_, suggesting that intracellular AA is converted to PGE_2_, which activates the cAMP/PKA pathway via EP2/EP4 receptors. Although HEK 293 cells and cerebellar granule neurons [22] express different types of PGE_2_ receptors, EP2 and EP4 are expressed in abundance by both cell types. Additionally, rNav1.4 currents and neuronal *I*_Na_ in the two cell types respond similarly to intracellular AA/PGE_2_. These data suggest that EP2 and EP4 play an important role in the regulation of sodium channels by intracellular AA/PGE_2_.

A previous study reported that sodium channels can be modulated by phosphorylation via the cAMP/PKA pathway because their α-subunits are the preferred substrates of PKA [25]. cAMP/PKA-dependent phosphorylation of rat brain voltage-gated sodium channels was reported to be restricted to five sites within the linker region between channel subunits I and II [32]. Although the sodium channel from rat skeletal muscle lacks this 210-amino acid linker region, its α-subunit is still reported to be an excellent substrate for PKA phosphorylation [26]. In this study, we investigated the specific amino acid residues that were responsible for modulation of rNav1.4 by intracellular cAMP and AA/PGE_2_. Based on our evaluation of the PKA phosphorylation sites predicted by Scansite, we generated two site-directed mutations in the N-terminal region of rNav1.4: rNa_V_1.4S56A and rNa_V_1.4T21A. rNa_V_1.4S56A current showed no significant change when cAMP was applied. Moreover, the AA/PGE_2_-induced increase in rNav1.4 current was abolished by the S56A mutation. When AA was applied intracellularly to HEK 293 cells expressing rNa_V_1.4S56A, current amplitudes were significantly inhibited, similar to the effect of extracellular AA on wild-type rNa_V_1.4 channels. These data suggest that AA and PGE_2_ can modulate sodium channels by different mechanisms, resulting in opposing effects. The rNa_V_1.4T21A mutation, on the other hand, did not completely eliminate the cAMP-induced increase in current.

The rNa_V_1.4T21A channel still responded to AA/PGE_2_ application despite elimination of the predicted T21 phosphorylation site. Altogether, our results indicate that although rNav1.4 lacks a linker region enriched with PKA phosphorylation sites, its S56 residue plays a pivotal role in intracellular AA/PEG_2_-mediated augmentation of rNa_V_1.4 current by activation of the cAMP/PKA pathway. These results are consistent with Yang and Barchi’s report, in which they detected rat skeletal muscle Na^+^ channel protein with [^32^P]phosphate labeling and forskolin stimulation, and found that phosphorylation via the PKA pathway was limited to serine residues [26].

Phosphorylation of ion channels causes changes in gating, resulting in acute increases and decreases in current [33]. In this study, we observed that although AA/PGE_2_ induced similar effects on neuronal *I*_Na_ and rNa_V_1.4 currents and activated the same pathways, their effects on the gating kinetics of steady-state activation were different in the two isoforms. AA/PGE_2_ increased neuronal *I*_Na_ amplitude by modulation of its steady-state activation [22]. However, neither steady-state activation nor steady-state inactivation was altered by intracellular AA/PGE_2_ in rNav1.4 channels. This difference might be due to the fact that the α subunits of neuronal Na^+^ channels such as Na_V_1.2 possess a loop I-II region that contains PKA phosphorylation sites, while rNa_V_1.4 does not possess this region [25]. In addition, the observed differences may be due to the fact that in our study, we did not co-transfect the rNav1.4 β subunit, an accessory protein of the sodium channel that may modulate its gating kinetics. Additional studies are needed to determine the role of S56 in rNa_V_1.4 channel activity and the role of rNav1.4 β subunits.

Na_V_ channels are not only responsible for the initiation and propagation of action potentials in a wide variety of excitable cells including skeletal muscle, but also contribute to many other cellular processes, including apoptosis, motility, and secretory membrane activity [34–36]. Diseases such as paramyotonia congenita and hyperkalemic periodic paralysis are examples of well-studied genetic ion channel diseases affecting skeletal muscle Na^+^ channels [37–39]. Massive AA release from membrane phospholipids and subsequent intracellular accumulation of free AA is a key cellular process in the control of inflammation and cell signaling [13]. A recent study indicated that heightened AA availability via supplementation enhanced endogenous PG synthesis and stimulated net skeletal muscle cell hypertrophy via a COX-2-dependent pathway [2]. Our study provides a new perspective to investigate the underlying mechanism of AA/PGE_2_-mediated regulation of skeletal muscle Na^+^ channels. Future work is needed to explore how AA/PGE_2_ may contribute to physiological or pathological abnormalities in skeletal muscle cells.
